# Altered gaze following during live interaction in infants at risk for autism: an eye tracking study

**DOI:** 10.1186/s13229-016-0069-9

**Published:** 2016-01-26

**Authors:** Emilia Thorup, Pär Nyström, Gustaf Gredebäck, Sven Bölte, Terje Falck-Ytter, The EASE Team

**Affiliations:** Uppsala Child and Baby Lab, Department of Psychology, Uppsala University, Uppsala, Sweden; BUP Stockholm, Center for Psychiatry Research, Stockholm County Council, Stockholm, Sweden; Karolinska Institutet Center of Neurodevelopmental Disorders (KIND), Pediatric Neuropsychiatry Unit, Department of Women’s and Children’s Health, Karolinska Institutet, Stockholm, Sweden

**Keywords:** Autism, Gaze following, Joint attention, Early development, Neurodevelopmental disorders, Social cognition, Communication, Younger siblings

## Abstract

**Background:**

The ability to follow gaze is an important prerequisite for joint attention, which is often compromised in children with autism spectrum disorder (ASD). The direction of both the head and eyes provides cues to other people’s attention direction, but previous studies have not separated these factors and their relation to ASD susceptibility. Development of gaze following typically occurs before ASD diagnosis is possible, and studies of high-risk populations are therefore important.

**Methods:**

Eye tracking was used to assess gaze following during interaction in a group of 10-month-old infants at high familial risk for ASD (high-risk group) as well as a group of infants with no family history of ASD (low-risk group). The infants watched an experimenter gaze at objects in the periphery. Performance was compared across two conditions: one in which the experimenter moved both the eyes and head toward the objects (Eyes and Head condition) and one that involved movement of the eyes only (Eyes Only condition).

**Results:**

A group by condition interaction effect was found. Specifically, whereas gaze following accuracy was comparable across the two conditions in the low-risk group, infants in the high-risk group were more likely to follow gaze in the Eyes and Head condition than in the Eyes Only condition.

**Conclusions:**

In an ecologically valid social situation, responses to basic non-verbal orienting cues were found to be altered in infants at risk for ASD. The results indicate that infants at risk for ASD may rely disproportionally on information from the head when following gaze and point to the importance of separating information from the eyes and the head when studying social perception in ASD.

**Electronic supplementary material:**

The online version of this article (doi:10.1186/s13229-016-0069-9) contains supplementary material, which is available to authorized users.

## Background

A father meets the eyes of his baby daughter and then looks toward a colorful toy sitting on the table. The baby follows his gaze and discovers the toy. Happily, she grabs it and smiles at her father. The scenario describes what is referred to as joint attention, the sharing of attention between individuals toward a common object. The ability to engage in joint attention is thought to play a critical role in socio-communicative development (e.g., [1, 2]). The fact that the baby in the example above is able to follow her father’s gaze allows the two of them to align their attention toward the toy. From this, the baby will eventually learn that others’ gaze direction is likely to be a cue to what is going on in their minds. It also allows for the father to tell his daughter the name of the toy and for her to learn a new word. Thus, the ability to attend to what others attend to forms a basis for later development in social cognition and language. Therefore, children who do not engage in joint attention might display altered development in these areas.

Typically developing infants robustly respond to other people’s gaze shifts before their first birthday [3, 4]. Considering the important role of joint attention in development, an early alteration in this function has been proposed as a possible common factor behind many of the various socio-communicative impairments displayed by children with autism spectrum disorders (ASD) (e.g., [5, 6]). Indeed, many children with ASD engage less in joint attention behaviors than their typically developing peers (e.g., [7, 8]). However, whether this reflects an early disruption in gaze following is not yet fully determined.

ASD is rarely diagnosed before the age of 2–3 years; long after gaze following typically develops. In a family with one child diagnosed with ASD, the probability of an ASD diagnosis in a sibling is more than tenfold higher than in the general population [9, 10]. A large study that followed younger siblings of children with ASD from early infancy found that 19.5 % of the sample later received an ASD diagnosis of their own [11]. This can be compared to a prevalence of about 1 % in the general population [12]. Following younger siblings of children with ASD hence provides a unique opportunity to investigate the early development of children who might later display ASD-related symptoms (for a review of findings of studies of younger siblings, see [13]).

Previous research has failed to find clear-cut evidence of gaze following impairments in infants at risk or in infants who later received an ASD diagnosis. Bedford et al. [14] showed videos of a model turning her head (and eyes) to look at one of two objects. Using eye tracking, the authors found no group differences in terms of gaze following accuracy, i.e., the tendency to look at the object being attended to by the model. They did find, however, that 13-month-old infants who later showed socio-communicative difficulties or ASD spent less time looking at the attended object than typically developing infants did. Therefore, the authors suggested that the key difficulty in ASD may not be the ability to follow gaze per se but rather to understand the communicative meaning of other people’s gaze. Two other studies [15, 16] compared younger siblings of children with ASD to younger siblings of typically developing (TD) children (ages 14–33 months) on a range of behaviors, including response to joint attention. Both studies used the Early Social Communication Scale (ESCS) [17] and did not find any group differences on its joint attention measure. Assessing response to joint attention with the ESCS entails the use of multiple cues; the experimenter calls the child’s name and points and looks toward the target item. Presmanes et al. [18] studied the effect of different combinations of verbal and non-verbal cues. They found that younger siblings of children with ASD (mean age 15 months) were as accurate in gaze following as the infants in the control group when multiple types of cues were used simultaneously. However, when fewer cues were used in combination, the younger siblings were significantly less likely to follow gaze than control infants. The children in this study went through clinical assessment at 34 months, and a follow-up study [19] revealed that performance on the early gaze following task, together with a measure of communicative actions initiated by the child, predicted later ASD diagnosis. Taken together, these results suggest a greater reliance on the use of multiple cues in infants who are later diagnosed with an ASD.

The largely negative findings from the high-risk sibling studies may be surprising in the light of earlier research (e.g., [20, 21]) that indicates that older children with an ASD diagnosis do not spontaneously use eye gaze information as a cue to where others direct their attention. One issue that may complicate comparisons across studies is whether or not information from the head could be used as a directional cue. All of the infant sibling studies used a model who turned her head toward the items being looked at. The presence of a head movement might constitute a confound that renders it impossible to conclude whether infants who follow gaze do so based on information from the eye gaze direction or from the head movement. In an event-related potential (ERP) study with 6–10-month-olds, Elsabbagh et al. [22] used dynamic face stimuli where gaze was shifted either toward or away from the infant. The study revealed that typically developing infants showed different neurological responses to gaze shifts toward versus away from them. The responses of those who were later diagnosed with ASD however did not differ between conditions, suggesting that these children may be impaired at processing information from the eyes. This would be in line with evidence from the adult and adolescent literature, showing that individuals with ASD have greater difficulty interpreting social and emotional information from the eyes [23, 24] as well as spend less time looking at other’s eyes as compared to non-autistic controls [25–27]. It is not known how the ERP findings of Elsabbagh et al. [22] translate to behavioral performance, but they clearly motivate further study of the effect of eye versus head information for gaze following in infants at risk.

The studies reported thus far differ on another potentially important dimension as well. While some of them used video or picture stimuli, others assessed the infants’ performance during live interaction. The use of pre-recorded video stimuli is a common approach that has the advantage of allowing a high degree of experimental control. There has however lately been a call for the use of more naturalistic stimuli (e.g., [28–31]). It has been argued that the difference between passively viewing and actively taking part in interaction must be accounted for in studies of social cognition [32–34]. Recent studies indicate that there are differences in the way that humans look at other people when they see them live as compared to when observed as video stimuli [35, 36]. It has also been demonstrated that eye contact is more effective at attracting another’s attention in a live situation than through video [37]. A number of studies have examined how live interaction affects the brain. In an fMRI (functional magnetic resonance imaging) study with adults [38], it was demonstrated that engaging in live interaction (through video from inside the scanner) results in greater activation in brain areas associated with social cognition than does viewing recordings of interaction. Another adult study [39] showed that following another person’s gaze live also activates areas associated with social cognition. In a study of 6-month-olds [40], it was found that observing body movements live affects activity in sensorimotor areas differently than does viewing recordings of the same movements. Measuring live interaction thus seems to evoke different responses than using pre-recorded stimuli, on a neural as well as behavioral level.

The aim of the present study was to assess gaze following in infants at risk for ASD, applying two essential modifications compared to previous research: experimental manipulation of the availability of head information and assessment during live interaction between experimenter and infant. To achieve this, we conducted an eye-tracking experiment in which the infants watched an experimenter look at different objects to her/his left or right, either—as in previous research—by turning both the eyes and head toward the object (Eyes and Head condition) or by shifting eye gaze with the head held still (Eyes Only condition). As noted, while infants at risk for ASD are able to follow gaze when a head movement is included [14], other evidence suggest that they are less sensitive to eye direction [22]. Against this background, we expected a group by condition interaction effect with a greater performance reduction in the Eyes Only condition (relative to the Eyes and Head condition) in the high-risk (HR) group compared to the low-risk (LR) group.

## Methods

### Participants

A total of sixty-four 10-month-old infants participated in the study (final sample, after exclusion; for participant characteristics, see Table 1). Forty-seven infants (21 boys, 26 girls) were high-risk infants (HR group), all having at least one older full sibling with an ASD diagnosis. Seventeen infants (11 boys, 6 girls) were low-risk infants (LR group), having no familial history of ASD and at least one typically developing older full sibling. Data from additional eight infants (7 HR, 1 LR) was excluded due to not meeting the inclusion criteria for least number of valid trials (see “Data reduction and analysis” section) or due to technical errors. All infants were part of an ongoing longitudinal study (Early Autism Sweden, EASE; www.smasyskon.se) following infant siblings and controls from 5 months of age to 36 months. The HR group was recruited through advertisements, the project’s website, and clinical units. LR infants were recruited from a database of families who had indicated interest in participating in research with their infants. Both groups consisted primarily of infants from the larger Stockholm area. All infants were born full term (>36 weeks) and did not have any confirmed or suspected medical problems, including visual/auditory impairments. The developmental level of the infants was assessed using the Mullen Scales of Early Learning (MSEL) [41]. Socioeconomic status was based on family income and parental education level. There were no group differences between HR and LR infants for developmental level or socioeconomic status (see Table 1), and the diagnosis of the sibling with ASD was confirmed through inspection of obtained medical records. At least 70 % of all assessments of older siblings were based on the use of the Autism Diagnostic Observation Schedule (ADOS) [42] and/or the Autism Diagnostic Interview—Revised (ADI-R) [43].

**Table 1:** Participant characteristics by group (HR = high risk, LR = low risk), final samples (mean/SD)

Measure	HR *N* = 47	LR *N* = 17	Pairwise comparison (*p* value^a^)
Age (months)	10.25/0.45	10.27/0.58	0.93
MSEL^b^ total score	98.45/13.96	96.35/11.70	0.52
MSEL VR^c^	54.32/10.05	53.71/8.09	0.79
MSEL FM^d^	55.09/9.34	55.18/9.42	0.78
MSEL RL^e^	44.74/10.47	42.35/11.77	0.40
MSEL EL^f^	42.70/10.47	41.12/10.69	0.64
SES^g^	−0.06/0.85	0.14/0.84	0.29

^a^Mann–Whitney U test

^b^Mullen Scales of Early Learning

^c^Visual Reception Subscale

^d^Fine Motor Subscale

^e^Receptive Language Subscale

^f^Expressive Language Subscale

^g^Socioeconomic status calculated on the basis of parental education and income (equal weighting), expressed as a *z*-score (for this measure, *N* = 45 in the HR group since two of the families did not disclose this information)

The study was approved by the Regional Ethical Board in Stockholm, and all parents provided written informed consent. The study was conducted in accordance with the standards specified in the 1964 Declaration of Helsinki.

### Procedure and stimuli

**Fig. 1:**
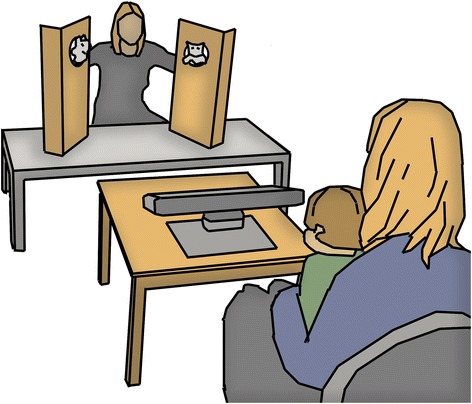
Sketch of the experimental setting. The infant and parent were seated at a distance of 200 cm from the experimenter. The visual angle of the experimenter’s face subtended 4.5° by 7°, and the holes where the puppets appeared each had a visual angle subtending 3.5° by 3.5°. A Tobii TX300 eye tracker (placed on a table in front of the infant) recorded the gaze of the infant. Two video cameras (not visible in the sketch) recorded the behavior of the infant as well as the stimulus area

The experiment was part of a more comprehensive assessment, and participants typically spent 4–5 h in the lab. The eye-tracking experiment was administered early during the visit, after a brief familiarization of the infant with the location and the involved staff. The MSEL was administered after the eye tracking. During the eye-tracking session, the infant was seated on the lap of the parent, at a distance of 200 cm from the experimenter (see Fig. 1). The experimenter was seated at a low table with two wooden screens mounted on top of it. Each screen had a hole at approximately the same level as the experimenters’ face. A Tobii TX300 eye tracker, placed on a table in front of the infant, recorded the infant’s gaze. Two video cameras recorded the behavior of the infant and the stimulus area. Before the session, a five-point calibration procedure was conducted. The experimenter moved a squeaky toy across predefined calibration points, making the toy emit a sound at each point to attract the attention of the infant. The procedure was repeated if necessary until calibration was satisfactory.

Repeated gaze following trials were embedded in a puppet show (lasting approximately 8–10 min in total), performed live while the infant’s gaze was recorded. The gaze following trials were presented in four blocks, separated by short playful breaks during which both experimenter and infant remained in their positions. Only gaze following data will be presented here.

At the start of each block, the experimenter attempted to attract the infant’s attention by looking at him/her, raising the eyebrows and vocalizing. The experimenter then used his/her hands to move two puppets behind the screens to make them appear in the holes. In order to make the infant look at the puppets, the experimenter moved them slightly back and forth while making a sound aimed at attracting the infant’s attention. During this period, the experimenter looked at the infant all the time and not toward the puppets. After this procedure, the first trial was presented.

Each trial started with the experimenter calling the infant’s name in order to elicit a gaze shift back to the experimenters’ face. If necessary, the name was called a second time, and if the infant still did not respond, the experimenter made a third attempt by making a funny face and a sound. Once the infant made eye contact, or after three unsuccessful attention bids had been administered, the experimenter shifted gaze toward one of the two puppets while making an excited vocalization (“Oj”—a Swedish interjection expressing surprise or excitement). The experimenter kept looking at the puppet for a total of 4 s before looking back at the infant. The experimenter then engaged in some baby-friendly small talk (e.g., commenting on the puppets without naming them) before continuing with the next trial.

The gaze following trials formed two experimental conditions as well as a third, exploratory condition. Since the third condition does not relate to the hypothesis, it will not be further described here but will be reported in the additional material (see Additional file 1).

In the Eyes and Head condition, the gaze shift toward the puppet was accompanied by a head turn. In the Eyes Only condition, the experimenter shifted eye gaze toward the puppet without turning the head; hence, the head was facing the infant but the eyes looked at the toy. The two conditions were presented sequentially within each block but counterbalanced across blocks. In order to reduce the procedural demands on the experimenter, who also needed to concentrate on keeping the infant’s attention; the left object was always attended first. Blocks 1 and 3 were identical (except for that in each block, a new type of puppet pair was used), with the conditions presented in the following order: Eyes and Head (left), Eyes and Head (right), Eyes Only (left), Eyes Only (right). Blocks 2 and 4 were also identical (except for a new type of puppet pair) and included Eyes Only (left), Eyes Only (right), Eyes and Head (left), and Eyes and Head (right), in this order. By this arrangement, we strived to achieve a balance between experimental control and feasibility. The puppets being used were toy rats (block 1), baby dolls (block 2), furry animals (block 3), and monkeys (block 4). The total duration of one block was around 60 s, with some variation depending on how fast the experimenter was able to catch the attention of the infant (for *M* and *SD*, see “Results” section).

To ensure that the results would not be due to a certain individual’s interaction style, and thus to aim for increased generalizability, the experiment was performed by 6 different individuals (2 males, 4 females). Four of them saw between 8 and 24 infants each. The proportion of HR versus LR infants did not differ between those experimenters. The two remaining experimenters only performed the experiment with HR infants (one and four each). The procedure was highly standardized to minimize the influence of the individual experimenters, and each experimenter was trained extensively to ensure that they adhered to the instructions. A video template of a whole session was used to train new experimenters.

### Data reduction and analysis

All recordings were manually coded by a lab member blind to the risk status of the infants as well as to the hypotheses. The coding was based on gaze replays (video of the stimulus area with the gaze of the infant superimposed) using the Tobii Studio 3.2.3 software (Tobii AB, Danderyd, Sweden).

We defined a time window that started when the experimenter engaged in direct gaze and ended when the experimenter shifted gaze from the puppet back to the infant. For a trial to be included in the analysis, the infant had to first fixate the experimenter’s face and then fixate one of the objects within this time window. Trials in which the infant, after the initial fixation at the experimenter, looked at the attended object were coded as *congruent*. Trials in which the infant moved his/her gaze from the experimenter to the unattended object were coded as *incongruent*. The gaze shift did not need to move directly from the experimenter to the target, as long as it did not pass the opposite target. Thus, trials in which fixations occurred on other parts of the stimulus area between the fixation on the experimenter and the puppet were still coded as either congruent or incongruent. Trials in which the infant looked at the face of the experimenter but did not continue to look at one of the puppets (i.e., the infant “got stuck” at the experimenter or looked away from the stimulus area) were coded as *other*. These trials were not included in the main analysis, but a group comparison on the number of *other* trials was carried out separately. All fixations shorter than 200 ms were excluded.

To be included in the analyses, each infant had to contribute at least two valid trials (25 %) per condition. This resulted in 64 infants (described above) being included in the Eyes and Head versus Eyes Only comparison.

The primary measure was a difference score (DS); the number of incongruent gaze shifts was subtracted from the number of congruent gaze shifts made by each infant. A positive DS hence indicates that the infant produced more congruent than incongruent gaze shifts. For a replication of the results using a proportional measure, see Additional file 2.

Since normal distributions could not be assumed, non-parametric tests were used throughout. Equal variances were confirmed for all variables. To test the hypothesis that our experimental manipulation affected the two groups differently, we subtracted the performance (the DS) in the Eyes Only condition from the performance in the Eyes and Head condition. Thus, the obtained measure reflects performance reduction in the Eyes Only condition relative to the Eyes and Head condition (positive values indicate reduction). This is analogous to testing the interaction effect in a 2 × 2 ANOVA with group and condition as factors [29]. Statistical analyses were performed in SPSS (SPSS Inc., Chicago, IL). Unless otherwise stated in the text, two-tailed probabilities were used.

## Results

### Preliminary analyses

The groups did not differ in terms of the total number of valid gaze shifts in either the Eyes and Head condition (HR *M* = 6.57, SD = 1.56; LR *M* = 6.24, SD = 1.95), *U* = 374.50, *p* = 0.69 or the Eyes Only condition (HR *M* = 6.45, SD = 1.57; LR *M* = 5.94, SD = 2.05), *U* = 356.00, *p* = 0.50 (Mann–Whitney *U* tests). Thus, the groups did not differ with regard to the amount of data produced in each condition, suggesting no group difference in terms of general attention. The number of trials coded as *other* (see “Methods” section) also did not differ between groups in either the Eyes and Head condition (HR *M* = 1.15, SD = 1.29; LR *M* = 0.94, SD = 1.09), *U* = 367.50, *p* = 0.61 or the Eyes Only condition (HR *M* = 1.60, SD = 1.56; LR *M* = 1.35, SD = 0.79), *U* = 396.50, *p* = 0.96. There was no group difference in the duration of trials (HR *M* = 59.02 s, SD = 12.99 s; LR *M* = 62.84 s, SD = 17.82 s), *U* = 364.00, *p =* 0.59. The time from when the experimenter started to engage in direct gaze to when s/he started shifting gaze toward the puppets also did not differ between groups (HR *M* = 5.81 s, SD = 1.55 s; LR *M* = 6.17 s, SD = 2.13 s), *U* = 378, *p* = 0.74, suggesting no differences in the time that had to be allocated to engaging the infants in eye contact.

No indication was found that the gaze following accuracy of the infants differed between individual experimenters. Since not all experimenters saw enough infants for a comparison between all experimenters to be meaningful, we compared the data from the experimenter who tested the largest amount of infants (38 %) to the combined data from the remaining experimenters. Mann–Whitney *U* tests on the performance reduction DS revealed no group differences in performance between experimenters on either the Eyes and Head condition, *U* = 448.00, *p* = 0.65, or Eyes Only condition, *U* = 478, *p* = 0.98, suggesting that our main findings cannot be explained by differences in interaction style between individual experimenters.

The gender distribution was somewhat different between groups. However, the performance did not differ between boys and girls in either the Eyes and Head condition (HR *U* = 261.00, *p* = 0.79; LR *U* = 22.00, *p* = 0.30) or the Eyes Only condition (HR *U* = 238.50, *p* = 0.45; LR *U* = 24.50, *p* = 0.40).

### Main results

**Fig. 2:**
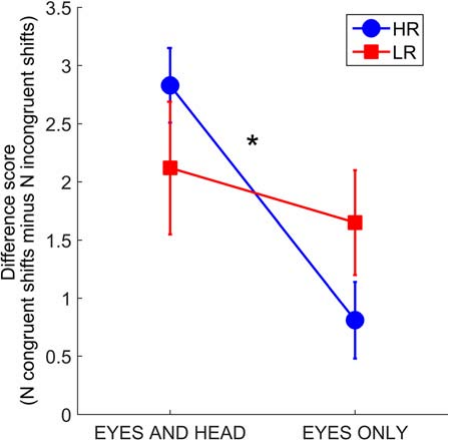
Main results. A significant group by condition interaction effect was observed, reflecting a performance reduction in the Eyes Only condition relative to the Eyes and Head condition in infants at risk for ASD, combined with similar performance in the two conditions in the low-risk infants. A difference score (DS; *y*-axis) was calculated for each group and condition by subtracting the number of incongruent trials from the number or congruent trials. *Error bars* represent standard errors

In line with our hypothesis, performance reduction when excluding head information was significantly larger in the HR group than the LR group (HR *M* = 2.02, SD = 2.68; LR *M* = 0.47, SD = 1.66), *U* = 240.00, *p* = 0.01, *r* = −0.31 ( Mann–Whitney *U* test; Fig. 2). This effect was followed up by comparisons on performance between conditions in each group as well as comparisons between groups in each condition. Bonferroni corrections for four comparisons were used. The analysis revealed that the HR infants were less likely to follow gaze in the Eyes Only as compared to Eyes and Head condition, *p* < 0.001, *r* = −0.65. The performance of the LR group did not differ between conditions, *p* > 0.99 (due to correction), *r* = −0.25 (related samples Wilcoxon signed rank tests). The accuracy difference scores did not differ between groups in either the Eyes and Head condition, *U* = 316.50, *p* = 0.80, *r* = −0.16, or the Eyes Only condition, *U* = 307.00, *p* = 0.62, *r* = −0.18.

A series of one-sample Wilcoxon signed rank tests were conducted to test whether the performance in the different groups and conditions differed from chance level (i.e., an accuracy difference score of zero). Both groups scored significantly above zero in both the Eyes and Head condition (HR *p* < 0.001; LR *p* = 0.003) and the Eyes Only condition (HR *p* = 0.008; LR *p* = 0.005).

As noted, results from the third, exploratory condition are reported in the additional material (Additional file 1).

## Discussion

This study shows that in an ecologically valid social situation—a live interaction between an infant and an adult who ostensibly indicates intention to communicate—responses to basic non-verbal orienting cues are altered in infants at risk for ASD. We found that the LR infants followed gaze as accurately in the Eyes Only as in the Eyes and Head condition. By contrast, a significant performance reduction in the Eyes Only condition compared to the Eyes and Head condition was found in the HR group. This demonstrates that gaze following is more affected by the head information in infants at risk for ASD than in infants at low risk for ASD (Fig. 2). Notably, both groups of infants were able to follow gaze above chance level in both conditions. Thus, eye information is sufficient to elicit gaze following in both groups.

The current study is, to our knowledge, the first to measure gaze following in infants at risk while experimentally assessing the role of eye direction versus head movement. We suspected that the head movement that usually accompanies the eye direction in similar designs could constitute a confound that makes it difficult to disentangle the role of the two types of information. Our results confirm the importance of this distinction and suggest that when children with or at risk for ASD previously have been found to follow gaze (e.g., [14, 44]), they might have been more reliant on the head movement than control children.

Although the HR group’s gaze following responses were more affected by head information than the LR group’s responses, it is notable that we did not observe a group difference in any of the two conditions when analyzed separately. Thus, it remains to be seen if the main results of this study reflect that the children at risk for ASD have attenuated gaze following when only information from the eyes is available, increased gaze following when head (and eye) cues are present, or both. We are not aware of any theoretical accounts for why the HR group could be expected to display higher gaze following accuracy in the Eyes and Head condition. In contrast, different views converge in the prediction of reduced responding in the HR group in the Eyes Only condition. First, children at risk for ASD may show selective impairments in processing information from the eyes. Interpreted from this perspective, our results are in line with those of Elsabbagh et al. [22] who found altered ERP’s in response to gaze cues in infants who later fulfilled diagnostic criteria for an ASD, as well as with the findings that individuals with ASD look less at eyes [25–27] and have difficulties interpreting eye information [23, 24]. Another possibility is that children at risk for ASD are less sensitive to directional cues in general and need more cues in order to respond typically. As noted in the introduction, Presmanes et al. [18] compared gaze following accuracy between HR and LR infants using different levels of cue combinations. A group difference was found only at the intermediate level of combinations (in that case vocalization + head/gaze shift). When multiple cues were used simultaneously, HR and LR infants were equally successful in responding to the gaze cues. When a silent head/gaze shift was the only cue, none of the groups displayed successful gaze following. There are several differences between the study by Presmanes and ours, one of them being that the infants in the Presmanes study were engaged in playing with a toy, whereas in our study, no toys were available and the experimenter made extensive attempts at catching the infant’s attention before starting the gaze following trials. More importantly, only the current study specifically addressed the role of head information for gaze following and whether eye information alone is sufficient to elicit accurate gaze following. Nevertheless, a common explanation could apply to both studies, namely that multiple simultaneous cues render gaze following easy for both groups, but that group differences arise when the number of cues is reduced.

Early alterations in joint attention are expected to influence later development in several domains. For example, in order to learn new words, children must be able to form an association between the object the adult is looking at and the word the adult is saying. Language development can therefore be expected to be negatively affected by poor gaze following. A study of 3-year-olds at high familial risk for ASD [45] showed that the ability to follow gaze was necessary but not sufficient for word learning to take place in the group of children displaying socio-communicative difficulties. The ability to engage in joint attention is also important in non-verbal communication and is thought to form a basis for later socio-cognitive development (e.g., [1, 2]). Given the crucial role joint attention plays in development, it is important to clarify the mechanisms underlying altered gaze following patterns in children with or at risk for ASD. Successfully doing so could enable targeted actions directed at improving gaze following in those at risk, which could positively affect later development on areas such as language and social cognition.

Whereas most eye-tracking studies show pre-recorded stimuli on a monitor, the current study assessed gaze following in a live setting. As accounted for in the introduction, previous studies [29, 35, 36] have shown that social attention measured using recorded materials is not necessarily comparable to social attention in real life. Conducting the experiment live has two distinct advantages relative to traditional video presentations [30]. First, it includes bi-directional contingent responding between the infant and experimenter. Second, it involves a “real” three-dimensional person, not a two-dimensional representation on the screen. Both of these aspects are key elements of human interaction and ensure a very high ecological validity of the current study relative to most previous work. We therefore believe that conducting the study live renders our results more likely to reflect performance in a real social situation.

To ensure that a high level of experimental control was obtained, we used eye tracking to attain precise measures of fixations and looking durations. This enabled a more exact coding procedure than if video coding alone had been used. In order to make sure that the experimenters did not treat the infants of the two groups differently, the experimental protocol was highly standardized and all experimenters were extensively trained. Indeed, analyses confirmed that the total length of the trials did not differ between groups and neither did the time that the experimenters devoted to engaging the infants in eye contact.

Our study indicates that gaze following based on eye information alone emerges earlier than typically assumed ([46], see also [47]). Specifically, Moore and Corkum [46] tested 18–19-month-olds using a design where the model shifted eye gaze while not moving the head. They found that only 18–19-month-olds followed gaze above chance level, leading them to conclude that the ability to follow gaze based on eye direction alone develops during the second year of life. There are a number of differences between that study and ours that might explain the differences in results. First, in the Moore and Corkum study, the target objects were placed outside the infant’s line of vision when facing forward. Moreover, the objects were only activated and made visible when the infant followed the experimenter’s gaze. Finally, the experimenter did not vocalize while looking at the target objects.

The current study compared infants at familial risk for ASD to infants with no familial history of the disorder. We do not yet know which of the infants will later receive an ASD diagnosis and which ones will display a typical course of development. However, all children will go through diagnostic assessment at 36 months and will then be compared based on diagnostic outcome rather than risk status. At that point, we will be able to conclude whether gaze following performance at 10 months will predict ASD diagnosis at an individual level. Another interesting line of research would be to follow up the current results at different time points to examine the development of gaze following, which is clearly not fully mature at 10 months in all infants. Doing so would allow us to distinguish whether the obtained difference will remain over time or whether it reflects a delay in the HR group. In order to find out whether altered gaze following specifically predicts ASD, future studies should aim to include other risk groups known to display comorbidity with ASD, e.g., infants at a high familial risk for ADHD.

A question that remains unanswered is whether motivational aspects can explain the altered performance of the HR group. It is possible that the social aspect of the experiment makes it more motivating for the LR infants and thus makes them more sensitive to the gaze cues. However, a lack of motivation could hardly explain the observed interaction effect. Also, no group differences were found in terms of the total number of valid trials in either condition.

The gender distribution in the sample was somewhat uneven across groups, with relatively more girls in the HR than LR group. However, since no effects involving gender were found, this is unlikely to have influenced the results. The two groups also differed in size, with the HR group comprised of 47 infants and the LR group of only 17. Importantly, the main result of the study reflected a statistically significant interaction effect. This analysis takes the number of participants in each group into account. Nevertheless, we had a different power to detect within-group effects in the two groups, which is a limitation. Of note, it is not uncommon in longitudinal designs to include more high-risk than low-risk children (e.g., [48]) partially because the HR children will be divided in multiple smaller groups after diagnostic assessment.

## Conclusions

Infants at familial risk for ASD were less likely to follow gaze when directional information from an adult’s eyes was available compared to when information from both eyes and head was available. This is in contrast to typically developing low-risk infants, whose gaze following performance did not differ between the two conditions. The results highlight the importance of separating information from the eyes from information from the head when studying gaze following in ASD/infants at risk.

## Additional files

Additional file 1:
**Description and discussion of a third condition that was not included in the main text.** (DOCX 18 kb) Additional file 2:
**The use and discussion of an alternative dependent measure.** (DOCX 13 kb)

## Abbreviations

ADI-R: Autism Diagnostic Interview—Revised
ADOS: Autism Diagnostic Observation Schedule
ASD: autism spectrum disorder
EASE: Early Autism Sweden
ERP: event-related potentials
ESCS: Early Social Communication Scale
fMRI: functional magnetic resonance imaging
HR: high risk
LR: low risk
MSEL: Mullen Scales of Early Learning
