# Supplementary material for: Altered gaze following during live interaction in infants at risk for autism: an eye tracking study
Source: Mol Autism. 2016 Jan 26;7:12. doi: 10.1186/s13229-016-0069-9 (PMC4729153; doi:10.1186/s13229-016-0069-9)
Supplement: Additional file 1: — Description and discussion of a third condition that was not included in the main text. (DOCX 18 kb) [file 13229_2016_69_MOESM1_ESM.docx]

**Additional Material**

**Additional file 1.**

**Methods**

For exploratory reasons, a third non-experimental condition labelled the No Object condition was included. This condition was not part of the counterbalancing; it consistently took place at the end of each block. Specifically, after the Eyes Only and Eyes and Head conditions were presented, the experimenter made the puppets disappear from the holes (accompanied by the same sound as when they appeared), by moving them downward behind the screens. The experimenter then turned the head to look at each empty hole once, making a vocalization intended to express surprise that the puppet was gone (Swedish: “Oj?”). Thus, this condition was similar to the Eyes and Head condition, with the exception that there was no obvious object to look at, only an empty hole. The rationale behind including this condition was that it could allow us to explore the role of the presence of a salient object in the gaze following context.

**Results**

For the Eyes and Head vs. No Object comparison, three infants were excluded from the HR-group for not having enough valid trials in the No Object condition. Thus, this comparison is based on data from 61 infants (44 HR, 17 LR).

Gaze following accuracy on the No Object condition (HR: *M* = 1.70, *SD* = 2.34; LR: *M* = 1.76, *SD* = 1.99) was compared to performance in the Eyes and Head condition (these conditions were similar in that they included head movements but differed in whether target objects were present or not). The statistical analysis followed the logic of the Eyes and Head vs. Eyes Only comparison. The performance reduction in the No Object condition as compared to Eyes and Head condition did not differ between groups, *U* = 336.50, *p* = 0.54, *r* = - 0.08 (Mann-Whitney U Test). Considering that the No Object condition was included for exploratory reasons, two Related Samples Wilcoxon Signed Rank Tests (Bonferroni corrected) were conducted to check for possible differences between conditions in the two groups separately. While the performance of the LR-group did not differ between conditions, *p* > 0.99 (due to correction), *r* = - 0.08, the HR-group had a higher gaze following accuracy in the Eyes and Head than the No Object condition, *p* = 0.004, *r* = - 0.48. One-Sample Wilcoxon Signed Rank Tests revealed that the performance of both groups was significantly higher than chance in the No Object condition (HR: *p* < 0.001; LR: *p* = 0.003). The groups did not differ significantly from each other in the No Object condition in terms of the DS, *U* = 359.00, *p* = 0.81, *r* = 0.03.

**Discussion**

The results concerning the Eyes and Head versus No Object comparison are inconclusive. No significant interaction effect was found. However, we did observe that whereas the LR-group performed comparably across conditions, the HR-group followed gaze more accurately when objects were present than when they were not. Together with our main result, this suggests that both head movements and the presence of salient targets enhance the performance of the HR-infants, while the LR-infants are less influenced by the addition of these cues. This is in line with the previous finding that high risk infants benefit more than low risk infants from the use of multiple cues [18]. If infants at risk for ASD are indeed less sensitive to eye information (as discussed in the main text), it is conceivable that the accumulation of cues is of greater importance for them than for infants who mainly rely on one type of cues (eye direction).
