# Supplementary material for: Altered gaze following during live interaction in infants at risk for autism: an eye tracking study
Source: Mol Autism. 2016 Jan 26;7:12. doi: 10.1186/s13229-016-0069-9 (PMC4729153; doi:10.1186/s13229-016-0069-9)
Supplement: Additional file 2: — The use and discussion of an alternative dependent measure. (DOCX 13 kb) [file 13229_2016_69_MOESM2_ESM.docx]

**Additional Material**

**Additional File 2**

We based our analyses on a difference score, since it has the advantage of preventing extreme scores based on few trials. Another option is to use a proportional measure, which has the advantage of controlling for the total number of valid trials affecting the results. To ensure that the results were not influenced by the choice of measure, the main analyses were re-run using the proportion of congruent gaze shifts out of all gaze shifts coded as congruent or incongruent. These analyses were based on 46 HR- and 17 LR-infants. The proportion congruent gaze shifts in the Eyes Only condition was subtracted from the proportion congruent gaze shifts in the Eyes and Head condition. A Mann-Whitney U Test revealed a significantly larger performance reduction in the HR- than LR-group, *U* = 233.00, *p* = 0.01, *r* = 0.31. In the HR-group, the gaze following accuracy in the Eyes and Head condition (*M* = 0.77, *SD* = 0.19) was significantly higher than in the Eyes Only condition (*M* = 0.61, *SD* = 0.23), *p* < 0.01, *r* = - 0.51. In the LR-group, the gaze following accuracy did not differ between the Eyes and Head condition (*M* = 0.69, *SD* = 0.26) and the Eyes Only condition (*M* = 0.72, *SD* = 0.24), *p* > 0.99 (due to correction), *r* = - 0.11 (Related Samples Wilcoxon Signed Rank Tests, Bonferroni-corrected). The results of the proportional measure analyses thus mirror the pattern revealed by the DS analyses.
